# Impact of maltodextrin and gum Arabic ratio on freeze dried microencapsulated extract of microgreen kangkong (*Ipomoea reptans* Poir)

**DOI:** 10.1038/s41598-025-19707-y

**Published:** 2025-10-14

**Authors:** Dian Kurniati, Karinanisa Adinda Rabani, Vira Putri Yarlina, Syamsul Huda, Rudy Adi Saputra

**Affiliations:** Department of Food Industrial Technology, Faculty of Agro-industrial Technology, Universitas Padjadjaran, Jatinangor, Sumedang, 45363 West Java Indonesia

**Keywords:** Bioactive compounds, Encapsulation, Gum arabic, Kangkong, Maltodextrin, Microgreen, Biotechnology, Plant sciences

## Abstract

Kangkong (*Ipomoea reptans* Poir) is widely consumed in Southeast Asia and possesses pivotal health benefits. Microgreen kangkong can be a potential alternative to meet nutritional requirements as it contains higher concentrations of bioactive compounds than mature plants. The study aimed to determine the effect of the ratio of maltodextrin and gum Arabic as encapsulants on the physicochemical characteristics of microgreen kangkong extract microcapsules. Encapsulation is applied to protect certain substances, including bioactive compounds, resulting in their good stability and bioavailability. Microgreen kangkong extract was encapsulated using the freeze-drying technique with varying concentration ratios between maltodextrin and gum arabic 60:40, 50:50, 40:60, and 20:80. The results showed that the increase in gum arabic concentration provided better chemical characteristics; however, this resulted in high water content. Extract with a concentration of maltodextrin and gum arabic 20:80 exhibited the foremost chemical characteristics than other treatments, with solubility, encapsulation efficiency, total phenol, total flavonoid, antioxidant, and anti-inflammatory activity of 97.35%, 86.85%, 82.70 mg GAE/10 g microcapsules, 43.42 mg QE/10 g microcapsules, 0.38 M/10 g microcapsules, and IC_50_ of 535.37 mg/L respectively. Based on the evaluation of both chemical and physical properties, the 20:80 maltodextrin to gum arabic ratio is considered as the optimal formulation due to its balance between bioactive retention and acceptable physical characteristics. This formulation demonstrates potential as a dry, bioactive-rich ingredient for functional food or nutraceutical applications and introduces microgreen kangkong as a novel source for microencapsulation matrices.

## Introduction

Kangkong (*Ipomoea reptans* Poir.) is a nutritional vegetable easily found in tropical regions that cover most of Africa, Asia, and Australia. Kangkong is rich in nutrients and has multiple benefits, such as maintaining liver function, preventing anemia, promoting heart and eye health, stabilizing cholesterol levels, reducing intestinal inflammation, and enhancing brain function^1^. Microgreens are young plants consisting of the first true leaves appearing or partially developing, with two cotyledon leaves that have fully developed. Microgreens can be planted in limited residential spaces with or without growing media, cultivated in confined spaces, and do not require excessive lighting. Furthermore, they are claimed to have higher bioactive compound concentrations, antioxidants, and anti-inflammatory properties than mature plants^2–4^.

Several types of microgreens studied, such as roselle, fennel, French basil, and sunflower, exhibit antioxidant potential composite index (APCI) and overall phytochemical composite index (OPCI) values that are higher or comparable to those of mature leaves^2^. However, there is still limited research on microgreens, especially on kangkong, which has a high potential for further study. While microgreens such as broccoli (*Brassica oleracea* var. italica) and fenugreek (*Trigonella foenum-graecum*) are more frequently reported in the literature, kangkong offers several unique advantages: (1) It is widely consumed and cultivated in Southeast Asia, making it culturally and economically relevant; (2) Its microgreen form contains significantly higher levels of phenolic compounds and antioxidant activity compared to its mature stage (Kurniati et al., 2024); and (3) It has a rich profile of bioactive compounds such as chlorogenic acid, rutin, and quercetin, which are beneficial for functional food applications^5,6^.

Due to the beneficial microgreen kangkong, the nutritional aspects must be protected to maintain important components and oxygen permeability in the matrix walls. One technique that can be utilized is encapsulation. Encapsulation techniques can extend the shelf life of materials from environmental influences by maintaining the stability of active compounds found in plants from degradation that can damage the material^7^. Microencapsulation technology has attracted significant interest in the food industry because of its ability to protect sensitive bioactive compounds, enhance the functionality of food products, and control the release and targeted delivery of encapsulated substances^8^. In this process, bioactive compound will be encapsulated using freeze-drying or lyophilization methods. This drying method is suitable for materials containing sensitive bioactive compounds because the drying process does not use high temperatures^9^. A recent study on mizuna (*Brassica rapa var. nipposinica*) microgreens demonstrated that encapsulated plays a crucial role in protecting and optimizing the bioavailability of easily degradable bioactive compounds^10^, as is also expected for microgreen kangkong. Similarly, research on broccoli microgreen juice encapsulated in maltodextrin using spray drying showed that this method effectively preserved phenolic content and maintained strong antioxidant activity, with the resulting powder proposed as a novel functional food ingredient. Microgreen kangkong likewise exhibits a rich profile of phenolics, antioxidants, and other phytochemicals at the micro stage, which are susceptible to degradation from oxidation, pH, and digestive enzymes^6,10,11^. Although the types and concentrations of bioactives differ among species, the protective mechanism provided by encapsulation remains both beneficial and applicable. Moreover, dried microgreens packed in capsule form have been recognized as a nutritional innovation that offers superior nutrient preservation, extended shelf life, portability, and precise dosage control, providing a convenient and efficient way to incorporate the full spectrum of microgreen nutrients into the daily diet^12,13^.

Maltodextrin and gum arabic were employed as encapsulating agents for the microgreen land kangkong extract. These carbohydrate-based encapsulants are used for their effectiveness and ease of application in microencapsulation. Maltodextrin has a high-water solubility, neutral taste and aroma, low viscosity, the ability to form a film layer, and protects the core material against oxidation. Gum arabic is a hydrocolloid that dissolves easily in water and acts as an excellent emulsifier to protect colloids, thereby complementing the performance of maltodextrin^14^. The study investigates the effect of the encapsulant ratio between maltodextrin and gum arabic on the physicochemical characteristics of microcapsules containing microgreen kangkong extract. The encapsulant ratio is expected to maintain or enhance the stability and bioavailability of bioactive compositions in microgreen kangkong, leading to significant bioactivity. This study is also expected to demonstrate that bioactive compounds, particularly those from encapsulated microgreen kangkong extract, offer numerous health benefits and can be applied as functional food ingredients in various functional food products. Despite its nutritional potential, the application of kangkong in microgreen or encapsulated functional food contexts remains underexplored compared to more commonly studied microgreens such as broccoli and fenugreek. By focusing on microgreen kangkong, which is widely consumed and culturally relevant in Southeast Asia, this study fills a notable research gap by providing novel data on its physicochemical properties and encapsulation efficiency, thereby contributing to the diversification of functional ingredient sources.

## Methods

### Materials

Microgreen kangkong (*Ipomoea reptans* Poir) was collected from Bandung Urban Agriculture Heritage (BUAH) The Living Lab (Indonesia). Gum arabic was purchased from TIC GUMS (Philadelphia, USA). Maltodextrin DE 10–12 was obtained from Qinhuandao Lihua Starch Co. (China). Sodium diclofenac, gallic acid, and quercetin were obtained from Sigma-Aldrich (St. Louis, Missouri, USA). Tris HCl was obtained from Himedia (India). Na_2_CO_3_, Bovine Serum Albumin (BSA), AlCl_3_, CH_3_COOK 1 M, FeCl_3_, FeSO_4_.7H_2_O, K_3_[Fe(CN)_6_].3H_2_O, KH_2_PO_4_, ethanol, methanol, NaOH 1 M, Folin-Ciocalteu reagent, Trichloroacetic Acid (TCA) were obtained from Merck (Rahway, New Jersey, USA).

### Sample preparation

**Fig. 1:**
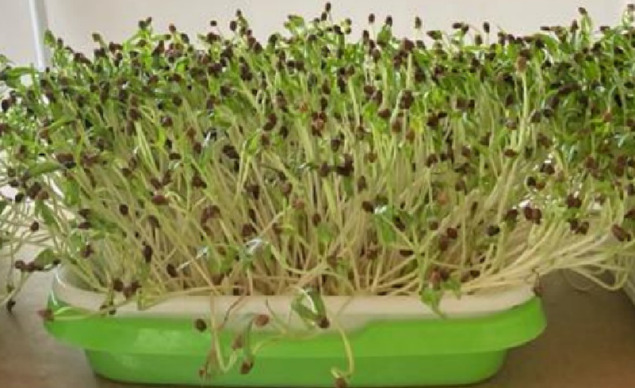
Nine-day-old microgreen kangkong at harvest time.

*I. reptans* P. microgreens were cultivated in a dark room without soil, provided with pure water to maintain humidity, and harvested nine days after cultivation (Fig. 1). After harvest, the plant was placed in clean and closed containers, then transported in a cool box to the laboratory. Microgreen kangkong was cleansed, air-dried, and cut into small pieces^15^. The fresh plant material was directly used for extraction without prior storage.

### Extraction of microgreen Kangkong

Microgreen kangkong was macerated in methanol 98% with a 1:10 ratio for 24 h. The macerate was filtered to obtain the methanol extract. Then, the residue from the maceration was soaked again using methanol for the second maceration. Subsequently, the extract solution was dried using a rotary evaporator (Buchi R-300, Swiss) at 50 °C and 40 rpm^16^.

### Encapsulation of microgreen Kangkong extract

**Fig. 2:**
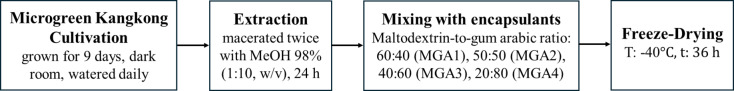
Flow diagram of experimental workflow.

Microgreen kangkong extract was dissolved with varying concentrations of maltodextrin and gum arabic (1:4), which was dissolved in a volume of 100 mL. Encapsulant ratios of maltodextrin and gum arabic were 60:40, 50:50, 40:60, and 20:80, marked as MGA1, MGA2, MGA3, and MGA4, respectively. Each treatment was ultrasonicated using an ultrasonic processor (Qsonica Q500, USA) for 15 min with 50% amplitude at 45 °C. The solution was dried using a freeze dryer (Christ Alpha 1–4 LD Plus, Germany) at -40 °C for 36 h^17^. All encapsulation treatments (MG1-MG4) were conducted in three biological replicates: each derived from separately prepared extract batches under identical conditions. Each biological replicate was subsequently analysed in triplicate to account for technical variation. Flow diagram of the experimental workflow is presented in Fig. 2.

### Yield

The yield is calculated based on the final product weight compared to the weight of the extract and encapsulant before drying^18^. The yield percentage can be calculated using the following formula:

1 $$\% ~Yield = \frac{B}{A} \times 100\%$$

A = Weight of the raw material used (g).

B = Final weight of the product (g).

### Water content

Water content was determined following AOAC^19^ (85.6% for microgreen kangkong). 0.5 g of encapsulant was dried using an oven (Wiseven, Korea) at 105 °C for 12 h. The sample was allowed to cool in a desiccator before being weighed. The water content percentage can be determined using the formula below:

2 $$\% ~Water~content = \frac{{B1 - B2}}{B} \times 100\%$$

B = Weight of the sample (g).

B1 = weight of sample + cup before drying (g).

B2 = weight of sample + cup after drying (g).

### Solubility

Solubility was carried out using the AOAC method^20^. 0.5 g of sample was mixed with 50 mL of distilled water and stirred using a magnetic stirrer for 5 min, then filtered with filter paper using a vacuum. The filter paper with residue is subjected to drying in an oven for 3 h at 105 °C. The drying process is carried out until a consistent weight is obtained. Solubility can be calculated using the following formula:

3 $$\% ~So\text{lub} ility = 1 - \frac{{c~ - ~b}}{{\left( {\frac{{100 - ka}}{{100}}} \right) \times a}} \times 100\%$$

a = weight of the sample (g).

b = weight of filter paper before use (g).

c = weight of filter paper after use (g).

ka = water content.

### Total phenolic content

Total phenolic content was measured following Kalpoutzakis et al.^21^. 1 ml of samples was mixed with 1.5 mL of Folin-Ciocalteu reagent and 3 mL of 7.5% Na_2_CO_3,_ followed by stirring for ± 1 min. Distilled water was added to adjust the final volume to 10 mL and thoroughly mixed. Then the mixture was incubated in a dark place for 30 min at room temperature. The absorbances were determined with a spectrophotometer (UV-9200 Beijing Rayleigh Analytical Instrument Corporation, China) at 765 nm. Total phenolic content is calculated as mg GAE/10 g microcapsules.

### Efficiency of encapsulation

**Fig. 3:**
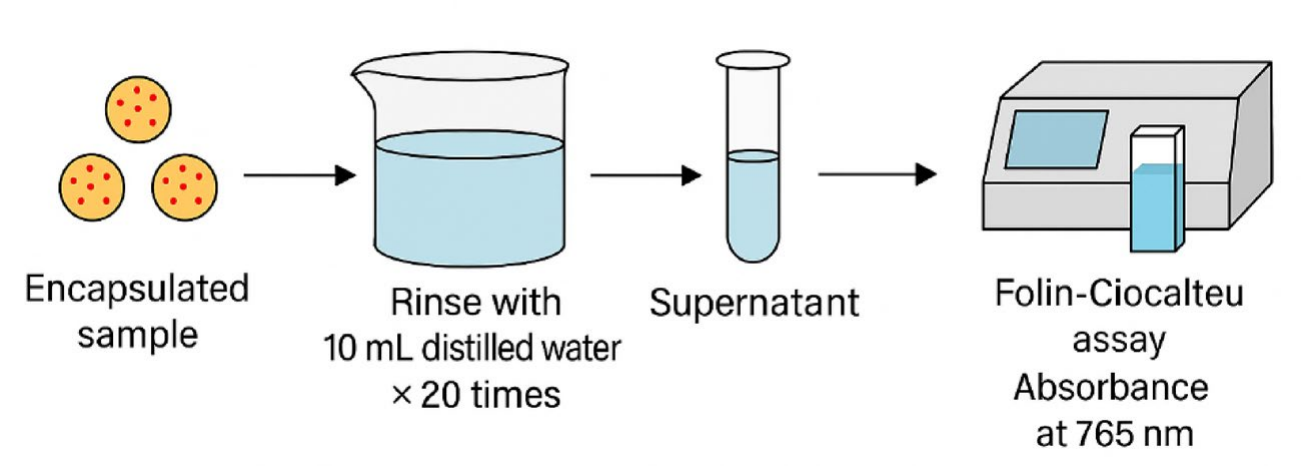
Visual schematic of quantification of surface polyphenol content (SPC).

Efficiency of Encapsulation (EE) was determined according to the Gibis et al.^20^ with slight modification. The total polyphenol content (TPC) and surface polyphenol content (SPC) were quantified with the same method described in the total polyphenol content section. To evaluate SPC, the encapsulated sample was gently rinsed with 10 mL of distilled water, repeated 20 times until the wash solution appeared clear. This step was intended to extract only the phenolic compounds that were either loosely attached or present on the surface of the microcapsules (Fig. 3). To quantify TPC, the encapsulated sample was extracted with methanol under stirring. The supernatants were then collected and subjected to the Folin-Ciocalteu assay to quantify SPC and TPC. The absorbances were recorded using a spectrophotometer (UV-9200 Beijing Rayleigh Analytical Instrument Corporation, China) at a wavelength of 765 nm. Efficiency calculation is determined by the following formula:

4 $$\% ~Encapsulation~Efficiency~(EE) = \frac{{TPC - SPC}}{{TPC}} \times 100\%$$

TPC = Total phenolic content (mg/L).

SPC = Surface phenolic content (mg/L).

### Morphological analysis

The morphological characteristics of the microcapsules was examined using Scanning Electron Microscopy (SEM, JEOL JSM-6360 LA, Japan) at 15 kV. The sample was mounted onto aluminum plate and coated with gold for 10–15 min at 8–10 mA. Representative micrographs of microcapsules were captured at a magnification of 1000x with a scale bar of 10 μm.

### Total flavonoid content

Total flavonoid content was determined as previously described by Nurhadi et al.^22^. 2 mL of samples were mixed with 0.2 mL CH_3_COOK 1 M and 0.2 mL of 10% AlCl_3_. The mixture was subsequently brought to a final volume of 10 mL with distilled water and gently stirred for 1 min. The mixture was incubated for 30 min at room temperature in a dark place. Absorbance was then recorded using a UV-Vis spectrophotometer at 431 nm. The total flavonoid content is calculated as mg QE/10 g microcapsules.

### Antioxidant activity

Antioxidant activity was quantified using the FRAP method, according to Derbel et al.^23^. 0.5 mL of samples (concentration variations of 2600, 2200, 1800, 1400, and 1000 mg/L) was added with 0.5 mL of phosphate buffer pH 6.6 (0.2 M) and 0.5 mL of 1% K_3_[Fe(CN)_6_].3H_2_O, followed by incubation at 50 °C for 20 min. Subsequently, 0.5 mL of 10% trichloroacetic acid (TCA) solution was added to form the upper layer. 0.5 mL of the upper layer was added by 0.5 mL of 0.1% FeCl_3_ and 0.5 mL of distilled water. The mixture was kept at room temperature for 5 to 10 min to react. Absorbance was then assessed using a UV-Vis spectrophotometer at 700 nm and converted to Fe^2+^ concentration (M/g) based on the Fe^2+^ standard curve. The formula for calculating antioxidant activity is as follows.

5 $$Antioksidan\left( {\frac{M}{g}} \right) = \frac{{Antooxidants~Concentration~\left( {\frac{{mol}}{L}} \right)}}{{1000}} \times DF \times Volume~(mL)$$

### Anti-Inflammatory activity

Anti-inflammatory activity was analyzed following Derbel et al.^23^. 1.5 mL of samples were added with 1 mL of 0.2% BSA in TBS and incubated for 30 min at 25 °C. The mixture was heated using a water bath (Nickel ElectroTM CliftonTM, England) for 5 min at 72 °C, then allowed to cool to room temperature. Absorbance was subsequently recorded at 660 nm using a UV-Vis spectrophotometer. The percentage of inhibition was determined using the following formula.

6 $$\% ~Inhibition = \frac{{Blank~absorbance - Sample~absorbance}}{{Blank~absorbance}} \times 100\%$$

A linear equation was obtained, and the IC_50_ value can be determined. The following is the formula for calculating the inhibition value (IC_50_).

7 $${\text{IC50 = }}\frac{{{\text{50 - a}}}}{{\text{b}}}$$

a = constant

b = intercept

## Results

### Yield of microgreen Kangkong extract encapsulation

**Fig. 4:**
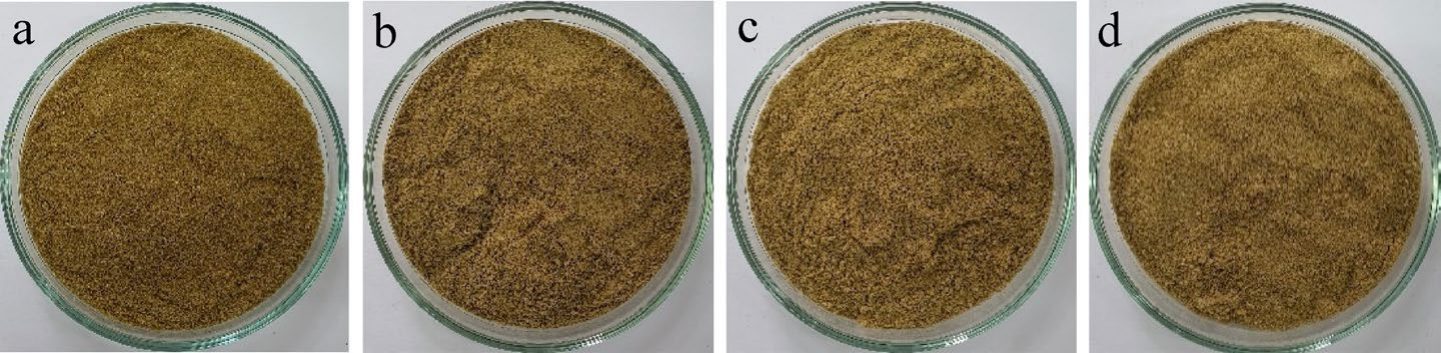
Visual appearance of the freeze-dried microgreen kangkong extract microcapsules prepared with different maltodextrin-gum arabic ratios: 60:40 (**a**), 50:50 (**b**), 40:60 (**c**), and 20:80 (**d**).

Yield is one of the important indicators in the production of a product. Several factors can influence the yield, such as the type and concentration of solvents, raw materials, and processing time during product manufacturing^24,25^. Yield calculation is based on the percentage ratio of the final weight (weight of the microcapsules produced) to the initial material (raw material used for encapsulation). Based on the data in Table 1, the yield values indicate significant differences among each treatment (*p* < 0.01), with the highest yield observed in MGA1. It is attributed to the highest maltodextrin concentration in the MGA1, which results in a higher yield than the other groups. Microgreen kangkong extract was successfully encapsulated using variation of maltodextrin and gum arabic ratio. The visual appearance of each encapsulated samples is shown in Fig. 4. The encapsulated microgreen kangkong formed a fine powder with a light brown colour. Increasing the concentration of gum arabic resulted in a lighter colour, which is consistent with previous studies showing that higher gum arabic content improves powder whiteness due to its good film-forming and emulsifying properties^26,27^.

**Table 1:** Physical characteristics of encapsulated microgreen Kangkong extract. Data represent mean ± sd from three biological replicates, each analyzed in triplicate. Different letters denote statistically significant differences according to duncan’s new multiple range test (DNMRT) at a 95% confidence level.

Treatment	Yield (%)	Water Content (%)	Solubility (%)
MGA1	58.32 ± 0.15^d^	3.73 ± 0.11^a^	98.73 ± 0.59^b^
MGA2	52.27 ± 0.35^c^	8.04 ± 0.26^b^	97.91 ± 0.52^ab^
MGA3	46.69 ± 0.36^b^	9.14 ± 0.07^c^	97.11 ± 0.89^a^
MGA4	34.24 ± 0.82^a^	9.98 ± 0.39^d^	97.35 ± 0.33^ab^

### Water content of encapsulated microgreen Kangkong extract

The water content was analyzed using a gravimetric method, where the sample was evaporated by heating and weighed until a constant weight was achieved. Water content is a crucial parameter in dry products such as powders to indicate the amount of moisture present in the material^28^. Water content values indicate significant differences among each treatment (*p* < 0.01) (Table 1). The lowest water content value was obtained from sample MGA1, which is 3.73%. The result indicated that as the maltodextrin concentration increased, the water content value decreased.

### Solubility of encapsulated microgreen Kangkong extract

Solubility is an essential parameter for dry products such as powders to demonstrate the product’s ability to dissolve in a solvent. Microcapsules can be considered acceptable if they exhibit high solubility in water^29^. The solubility values significantly differ for each treatment (Table 1). MG1 showed the highest solubility (98.73%), which was significantly higher than MG3 (97.11%) (*p* < 0.05). MG2 and MG4 had intermediate solubility values that were not significantly different from MG1 or MG3. This solubility value can be considered good as it approaches 100%, indicating high-quality microcapsules.

#### Encapsulation efficiency of encapsulated microgreen Kangkong extract

Encapsulation efficiency demonstrates the effectiveness of the encapsulant as a protector of the core material. A good encapsulating material exhibits high efficiency and effectively shields the material inside^30,31^. Encapsulation efficiency is based on the encapsulated phenolic content divided by the total phenolic content of the microcapsules overall. Determination of encapsulation efficiency values is performed using UV/Vis spectrophotometry, followed by calculations using regression equations obtained from the standard curve. The encapsulation efficiency values differed significantly among treatments (*p* < 0.01), indicating that the type and ratio of encapsulant influenced the protective ability of the microcapsules. It showed that the range of encapsulation efficiency values is 72–87% **(**Table 2**)**. MGA4 gave the highest encapsulation efficiency with a value of 86.85%.

**Table 2:** Encapsulation efficiency of encapsulated microgreen Kangkong extract. Data represent mean ± sd from three biological replicates, each analyzed in triplicate. Different letters denote statistically significant differences according to duncan’s new multiple range test (DNMRT) at a 95% confidence level.

Treatment	Encapsulation Efficiency (%)
MGA1	72.77 ± 1.34^a^
MGA2	78.40 ± 1.85^b^
MGA3	82.65 ± 1.51^c^
MGA4	86.85 ± 1.62^d^

#### Morphological analysis of encapsulated microgreen Kangkong extract

**Fig. 5:**
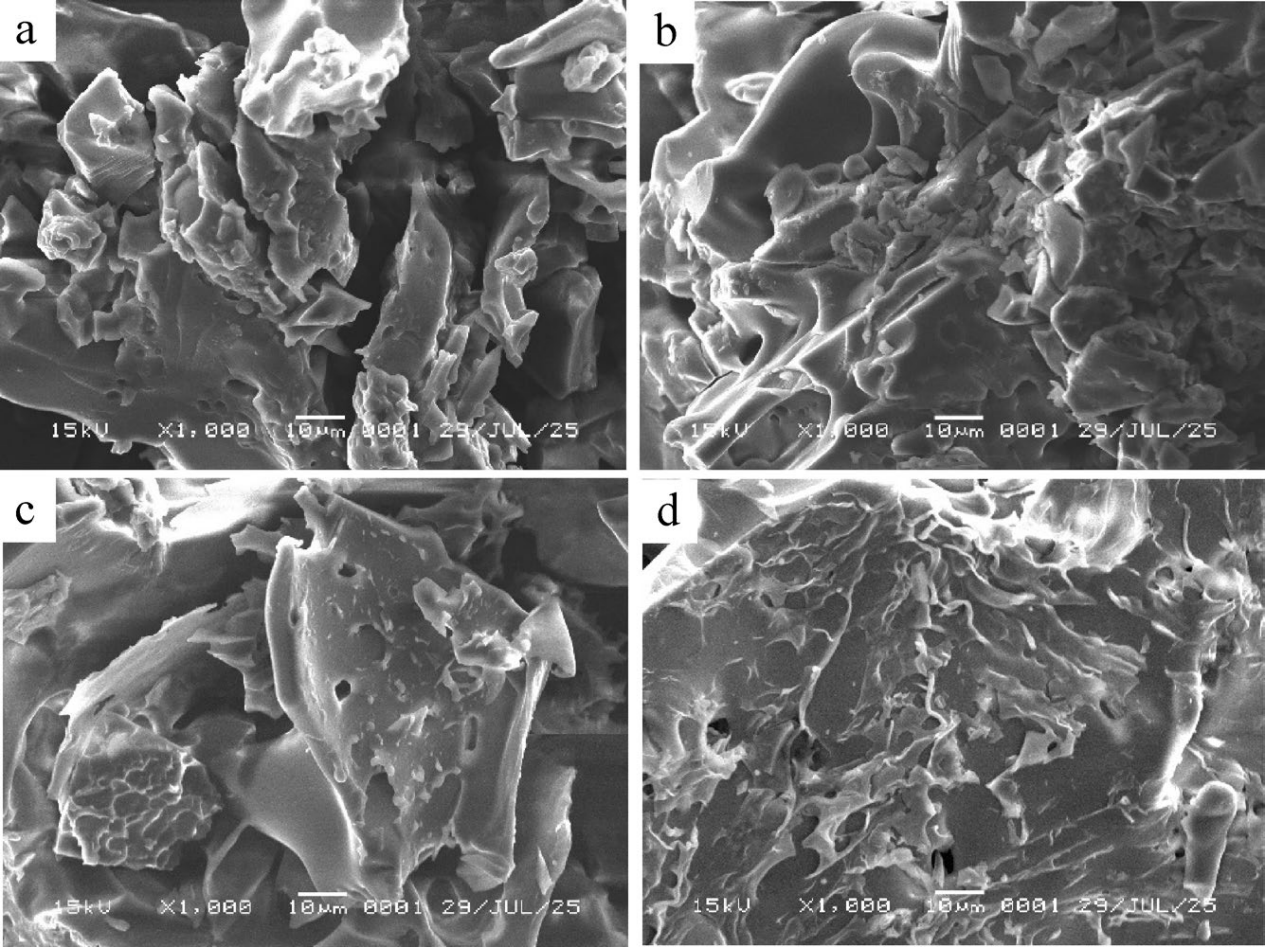
SEM images of the freeze-dried microgreen kangkong extract microcapsules prepared with different maltodextrin-gum arabic ratios: 60:40 (a), 50:50 (b), 40:60 (c), and 20:80 (d).

SEM images of the freeze-dried microgreen kangkong extract are presented in Fig. 5. The images revealed irregular, flake-like, and collapsed structures rather than smooth spheres, which is typical for freeze-dried products^32^. Microcapsules with the highest maltodextrin content (MGA1, 60:40) (Fig. 5a) exhibited dense structures with small, tightly packed pores. At intermediate maltodextrin ratio (MGA2, 50:50, and MGA3, 40:60) (Fig. 5b, c), surfaces showed more visible indentations and moderate porosity, reflecting a more open structure compared to MGA1. The formulation with the highest gum arabic content (MGA4, 20:80) (Fig. 5d) displayed a porous, sponge-like appearance with more pronounced surface collapse. likely due to gum arabic’s higher water retention capacity and viscosity, which influence ice crystal formation and sublimation during freeze-drying^32,33^.

### Total phenolic content of encapsulated microgreen Kangkong extract

**Fig. 6:**
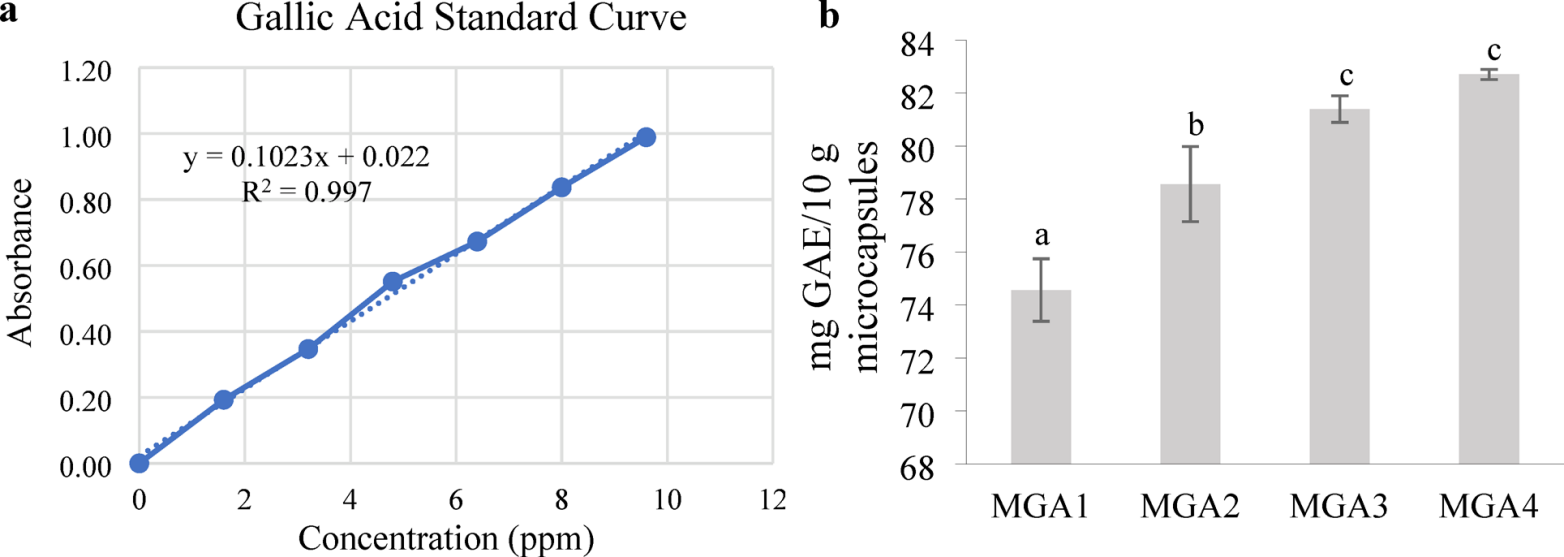
Standard curve of gallic acid (**a**) and total phenolic content of encapsulated microgreen kangkong extract (**b**). Data represent mean ± SD from three biological replicates, each analyzed in triplicate. Different letters indicate significant differences based on Duncan’s New Multiple Range Test (DNMRT) at a 95% confidence level.

The total phenolic content was measured based on mg gallic acid equivalents per 10 g of microcapsules (mg GAE/10 g microcapsules). The range of total phenolic content of microgreen kangkong extract can be seen in Fig. 6. Total phenolic content increases with higher gum arabic concentrations. However, MGA3 and MGA4 do not exhibit significant differences in total phenolic content, with the highest value observed in MGA4 at 82.7 mg GAE/10 g microcapsules.

### Total flavonoid content of encapsulated microgreen Kangkong extract

**Fig. 7:**
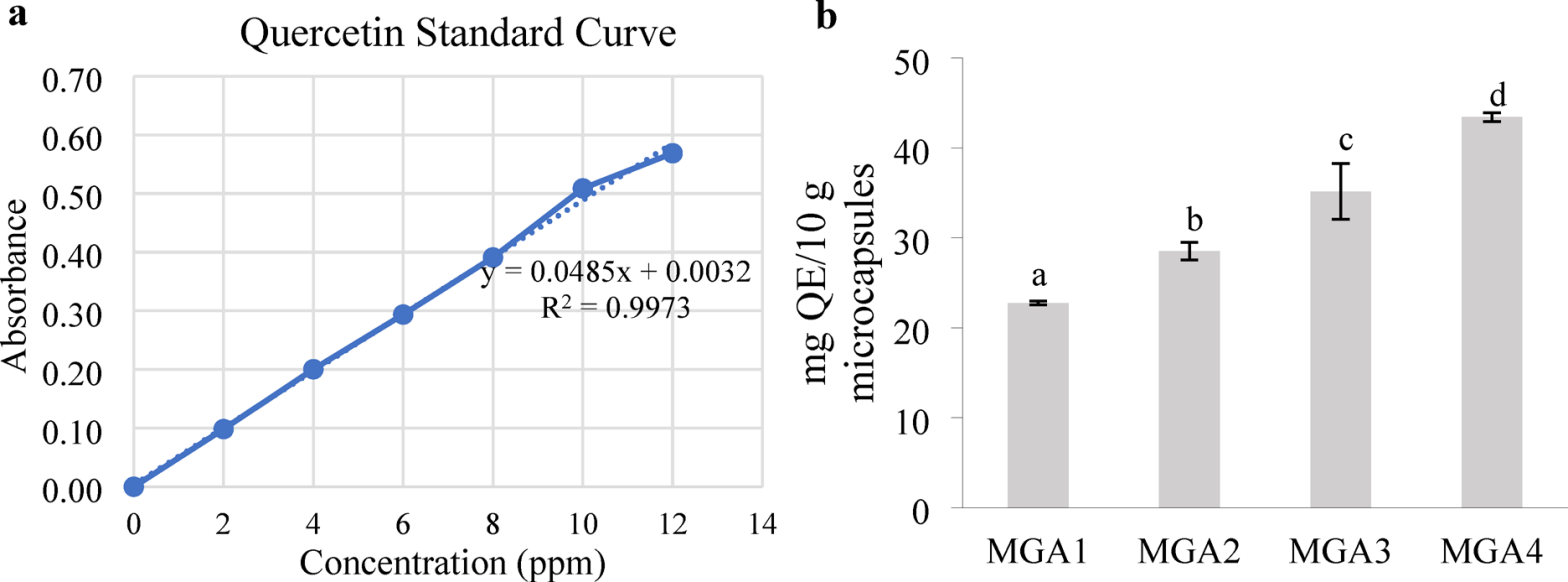
Standard curve of quercetin (**a**) and total flavonoid content of encapsulated microgreen kangkong extract (**b**). Data represent mean ± SD from three biological replicates, each analyzed in triplicate. Different letters indicate significant differences based on Duncan’s New Multiple Range Test (DNMRT) at a 95% confidence level.

Total flavonoid content was expressed in mg quercetin equivalents per 10 g of microcapsules (mg QE/10 g microcapsules). Flavonoid content shows a marked difference between each treatment (*p* < 0.01) (Fig. 7**)**. MGA1 significantly differs from MGA2, MGA3, and MGA4, with the highest total flavonoid content found in MGA4 at 43.42 mg QE/10 g microcapsules. These results demonstrate that the total flavonoid content increases with the increasing concentration of gum arabic as the encapsulant.

### Antioxidant activity of encapsulated microgreen Kangkong extract

**Fig. 8:**
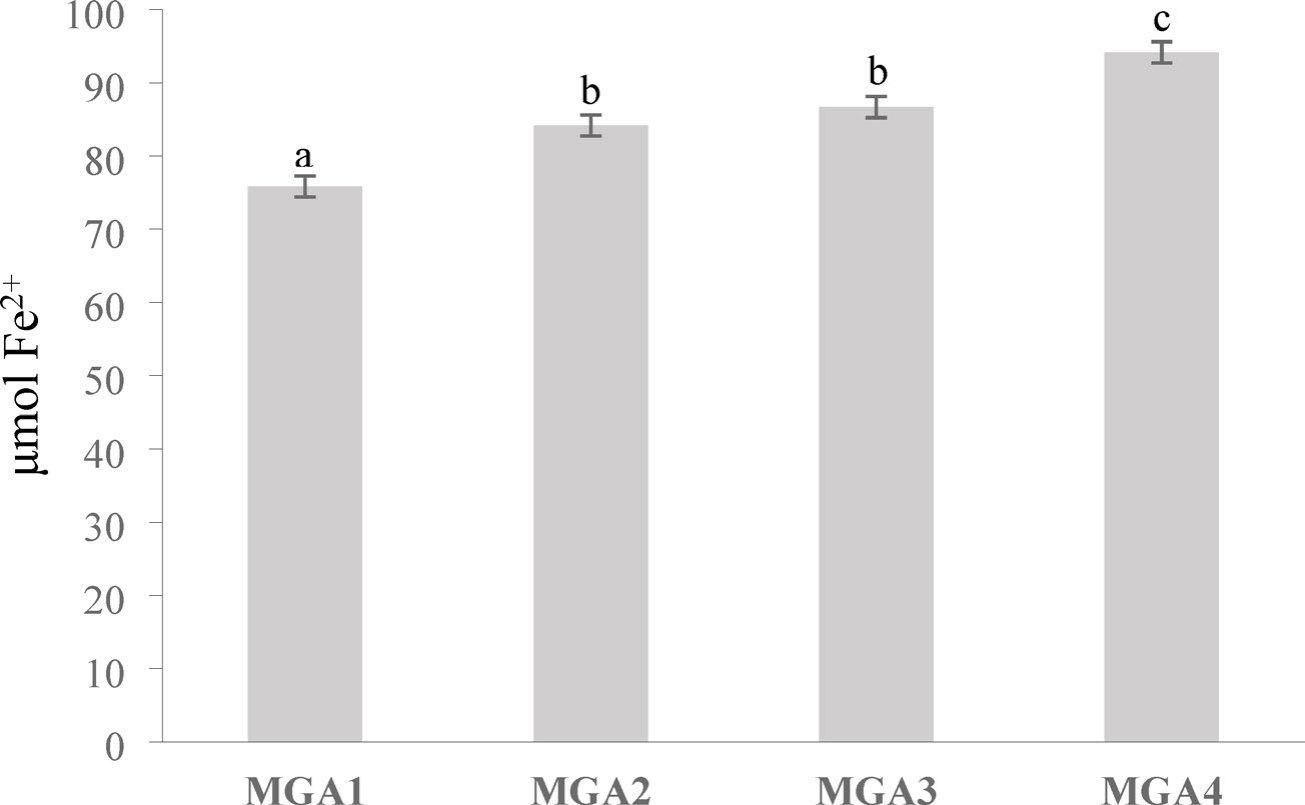
Antioxidant activity by Ferric reducing antioxidant power (FRAP) assay of encapsulated microgreen kangkong extract. Data represent mean ± SD from three biological replicates, each analyzed in triplicate. Different letters indicate significant differences based on Duncan’s New Multiple Range Test (DNMRT) at a 95% confidence level.

The antioxidant activity reflects the ability of sample to neutralize free radicals and prevent oxidative damage. Antioxidant activity is measured using the FRAP method, which determines the sample’s capacity to reduce Fe³⁺ ions to Fe²⁺ ions^34^. The antioxidant activity showed significant differences between treatments, with the highest antioxidant activity value observed in MGA4 at 94.17 µmol Fe^2+^ **(**Fig. 8**)**. This value was significantly higher than those observed in the other treatments (*p* < 0.01), whereas MGA2 and MGA3 showed intermediate antioxidant activities without significant differences between them. The results indicate that increasing the concentration of gum arabic enhances antioxidant activity.

## Anti-Inflammatory activity of encapsulated microgreen Kangkong extract

**Fig. 9:**
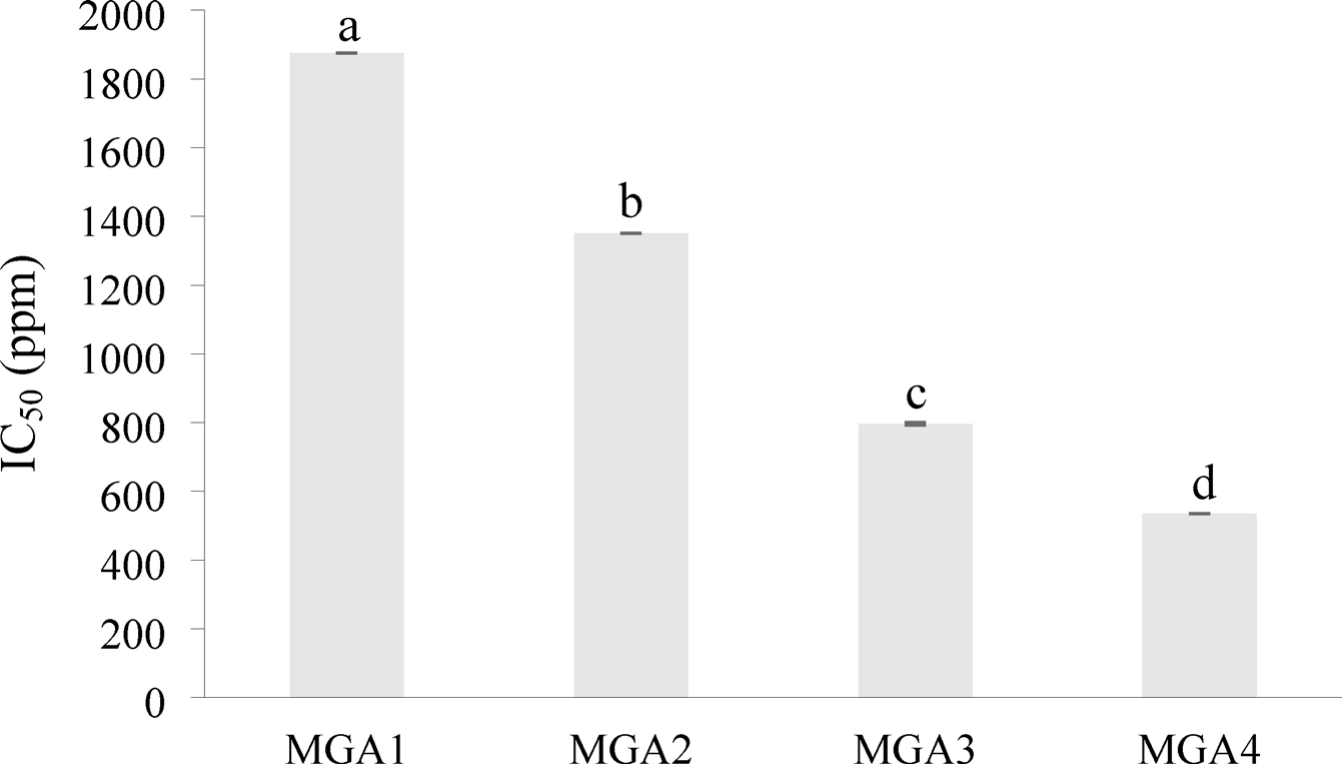
Anti-inflammatory activity of encapsulated microgreen kangkong extract. Data represent mean ± SD from three biological replicates, each analyzed in triplicate. Different letters indicate significant differences based on Duncan’s New Multiple Range Test (DNMRT) at a 95% confidence level.

The anti-inflammatory activity was determined using the BSA method based on its inhibition against protein denaturation, a parameter indicating inflammation. The anti-inflammatory activity was expressed as the IC_50_ value (ppm), determined using a standard curve with sodium diclofenac as the positive control. The highest anti-inflammatory activity was observed in MGA4 treatment, with an IC_50_ value of 535.37 ppm **(**Fig. 9**)**. The results indicate that increasing the concentration of gum arabic leads to a decrease in the IC_50_ value, thereby resulting in high anti-inflammatory activity.

## Discussion

The utilization of high concentrations of maltodextrin produces the best physical characteristics of microcapsules, producing the highest yield, lowest water content, and good solubility. Maltodextrin, a filler material, has a high solubility, influencing the increase in total dissolved solids, thereby enhancing the yield obtained^35^. According to Ribeiro et al.^36^, the highest maltodextrin content showed a higher yield of carotenoid-rich microcapsules. This aligns with findings in elderberry pomace microencapsulation, where formulations using higher maltodextrin proportions achieved encapsulation yields of 75–84% with robust solubility and protective matrix effects^37^.

The difference in concentration between maltodextrin and gum arabic also markedly affects the water content in microcapsules, which is caused by the differences in weight and molecular structure between them. In this study, increasing the percentage of gum arabic in the encapsulation ratio caused high water content. The highest water content observed was 9.98% in the MGA4 (20:80) treatment. This moisture level is considered acceptable and safe for powdered extract, as maintaining water content below 10% is widely recognized to enhance product stability and reduce risk of microbial contamination^38,39^. Moreover, the water content in MGA4 was significantly different from all other treatments (*p* < 0.01), indicating that the variation in maltodextrin and gum arabic ratios influenced the moisture retention of the powders. This is consistent with findings by Fishi et al.^40^, who reported that formulations with higher gum arabic content showed increased moisture levels due to gum arabic’s greater hydrophilicity and its ability to retain water molecules more effectively than maltodextrin. Maltodextrin has a molecular weight of less than 4,000. In contrast, gum arabic has a larger molecular weight, approximately 500,000, resulting in stronger molecular bonds between water and gum arabic, making it more difficult for water to evaporate during drying. Additionally, gum arabic has high viscosity^41^. Low water content in materials is often associated with low water activity. The water content can also affect the solubility of a substance in water. High water content in a substance prevents it from absorbing large amounts of water, making it difficult to disperse in water due to its sticky nature, thus limiting the formation of pores to a maximum extent^42^. Additionally, substances with high water content typically have large particle sizes and tend to clump together, reducing the surface area for wetting. The solubility of a product can also be influenced by the type and concentration of fillers or binders used during the manufacturing process. Gum arabic and maltodextrin have high water solubility, contributing to the high solubility values. All treatments exhibited solubility values ranging from 97.1 to 98.7%, which are considered good as they approach 100%, indicating high-quality microcapsules. These results are comparable to those reported by Mutavski et al.^37^ who achieved solubility values between 95% and 98% for microcapsules produced using maltodextrin and gum arabic to encapsulate elderberry by product extract. Overall, both studies demonstrate that maltodextrin and gum arabic effectively produce microcapsules with excellent water solubility, which is essential for their application in functional foods.

SEM observations showed that all freeze-dried microcapsules exhibited irregular, flake-like, and partially collapsed surfaces—morphologies typical of freeze-dried products due to ice crystal sublimation. Maltodextrin-rich formulations appeared denser with fewer pores, whereas gum arabic-rich formulations, particularly MGA4, showed more porous and sponge-like structures. This greater porosity in gum arabic-rich systems has been associated with enhanced phenolic retention and antioxidant preservation, attributed to gum arabic’s higher viscosity and emulsifying capacity^36,39^, indicating that microstructural differences might influence the functional quality of the powders^37,43^.

Microcapsules with higher concentrations of gum arabic exhibited the best chemical properties, including encapsulation efficiency, total phenolic content, flavonoid content, antioxidant activity, and anti-inflammatory activity. The type of encapsulant significantly affects encapsulation efficiency. Gum arabic is often used as an encapsulant due to its ability to form layers/films and good emulsifying properties^26^. Gum arabic can maintain the stability of active compounds by increasing the viscosity of sulution; thus, higher concentrations of gum arabic enhance viscosity, promoting stronger intermolecular interaction that improve the encapsulation process^44^. Encapsulation efficiency in this study ranged from 72 to 87% and was significantly affected by the encapsulant type and ratio (*p* < 0.01). The MGA4 treatment, with a higher gum arabic content, exhibited the highest efficiency (86.85%), demonstrating the importance of gum arabic in effectively protecting bioactive compounds. Similarly, Tonon et al.^39^ reported that increasing gum arabic concentration improved encapsulation efficiency and powder stability by forming a more cohesive protective film around bioactive compounds during spray drying.

Gum arabic belongs to the polysaccharide class and contains a small amount of covalently bound protein, where the effective interaction between polysaccharides and proteins in emulsions between materials can enhance physical and oxidative stability that protects flavonoid content from oxidation and degradation^45^. Gum arabic can protect core materials from detrimental changes because its binding properties enable the formation of effective films and emulsions^46^. The research conducted by Mohsin et al.^47^ on the encapsulation of kombucha using 10% gum arabic could sustain its antioxidant activity. Antioxidant activity is also associated with its total phenolic and flavonoid contents. In this study, MGA4 exhibited the highest total phenolic and flavonoid contents, correlating with its superior antioxidant activity. These values were significantly higher than those of other treatments (*p* < 0.01), indicating that higher gum arabic concentration enhances the retention of bioactive compounds and antioxidant capacity in the microcapsules. According to Nurhadi et al.^48^, encapsulating dragon fruit extract formulated with higher gum arabic proportions showed a 15–20% increase in total phenolic contents retention compared to maltodextrin-only formulations. Similarly, Ribeiro et al.^36^ reported that a maltodextrin-gum arabic ratio of 30:70 not only preserved phenolic compounds but also flavonoids, demonstrating significantly improved stability compared to formulations using maltodextrin alone. Using gum arabic as an additional matrix enhances material stability and protects it from oxidation, effectively protecting the material’s total phenolic and flavonoid content^49^. Gum arabic has a highly branched structure capable of forming bonds that protect bioactive content in materials^50^. According to Al-Jaber et al.^51^, phenolic compounds, alkaloids, terpenoids, and organic sulphur components are bioactive compounds that act as natural antioxidants. However, antioxidant activity is not always correlated with phenol or flavonoid content due to other influencing factors such as variations in active components in plants, antagonistic or synergistic effects among the active components, and the methods and conditions used during the study^52^.

Encapsulation protects active compounds acting as anti-inflammatory agents, including polyphenols. Polyphenol compounds have anti-inflammatory effects by reducing the production of pro-inflammatory molecules and aiding a balanced immune response, thereby modulating inflammation pathways. Polyphenols also play a role in preventing and treating inflammatory diseases and reducing chronic inflammation^53^. Moreover, flavonoids, a group of polyphenols, are known for their anti-inflammatory properties. They inhibit protein denaturation and interact with albumin to stabilize protein structures. The interaction involves hydrogen bonding between the N atom in amino acid residues, the O atom in the carbonyl group of flavonoids, and hydrogen atoms in the hydroxyl groups of flavonoids^54^. The anti-inflammatory activity results highlight the protective role of encapsulation in preserving bioactive compounds. Using the BSA method, the MGA4 treatment exhibited the highest activity with the lowest IC_50_ value of 535.37 ppm, indicating stronger inhibition of protein denaturation This value was significantly different from those of other treatments (*p* < 0.01), demonstrating the impact of gum arabic concentration on enhancing anti-inflammatory effects. Increasing gum arabic content also improves encapsulation efficiency and stabilizes polyphenols and flavonoids, thereby boosting their antioxidant and anti-inflammatory properties by providing a protective matrix that reduces degradation during processing and storage. Recent studies on land spinach support these findings, showing that a maltodextrin-to-gum arabic ratio of 20:80 effectively preserves polyphenols and maintains anti-inflammatory activity during storage, underscoring the key role of gum arabic in protecting bioactive compounds^55^. These findings also align with other reports emphasizing the effectiveness of gum arabic-based encapsulation in maintaining the bioactivity of plant-derived compounds^37,56^.

## Conclusion

The ratio of maltodextrin and gum arabic significantly affects the physicochemical characteristics of microcapsules containing microgreen kangkong extract. A higher proportion of gum Arabic (20:80) in the encapsulant resulted in the finest chemical properties, including encapsulation efficiency, total phenolic content, flavonoid content, antioxidant activity, and anti-inflammatory properties. Increased gum arabic utilization demonstrated good stability but also caused an increase in water content, which is a crucial parameter in dry products. Overall, the 20:80 maltodextrin to gum arabic ratio was identified as the optimal formulation, offering a balance between bioactive compound retention and acceptable physical properties. This optimized formulation demonstrates potential for application in functional food or nutraceutical products.

## Data Availability

All data generated or analyzed during the study are included in this published article.
